# Computer-Generated Ovaries to Assist Follicle Counting Experiments

**DOI:** 10.1371/journal.pone.0120242

**Published:** 2015-03-26

**Authors:** Angelos Skodras, Gianluca Marcelli

**Affiliations:** 1 German Centre for Neurodegenerative Diseases (DZNE), Tübingen, Germany; 2 School of Engineering and Digital Arts, University of Kent, Canterbury, United Kingdom; Center for Human Reproduction, UNITED STATES

## Abstract

Precise estimation of the number of follicles in ovaries is of key importance in the field of reproductive biology, both from a developmental point of view, where follicle numbers are determined at specific time points, as well as from a therapeutic perspective, determining the adverse effects of environmental toxins and cancer chemotherapeutics on the reproductive system. The two main factors affecting follicle number estimates are the sampling method and the variation in follicle numbers within animals of the same strain, due to biological variability. This study aims at assessing the effect of these two factors, when estimating ovarian follicle numbers of neonatal mice. We developed computer algorithms, which generate models of neonatal mouse ovaries (simulated ovaries), with characteristics derived from experimental measurements already available in the published literature. The simulated ovaries are used to reproduce *in-silico* counting experiments based on unbiased stereological techniques; the proposed approach provides the necessary number of ovaries and sampling frequency to be used in the experiments given a specific biological variability and a desirable degree of accuracy. The simulated ovary is a novel, versatile tool which can be used in the planning phase of experiments to estimate the expected number of animals and workload, ensuring appropriate statistical power of the resulting measurements. Moreover, the idea of the simulated ovary can be applied to other organs made up of large numbers of individual functional units.

## Introduction

Accurate estimation of ovarian follicle numbers is the foundation of reproductive biology [1]. Follicle counts are important for the comparison between wild-type animals and those carrying specific genetic mutations that affect the reproductive system [2], the determination of the adverse effects of environmental toxins [3] and cancer chemotherapeutics [4], as these factors may affect the number of follicles within the ovaries. Furthermore, precise follicle counts are required when studying the developmental progress of the ovarian follicles, their quiescent state, their recruitment and loss thereof [5 and 6]. The number of follicles in an ovary of an animal can be considered as a statistical variable that follows a probability distribution. This distribution is representative of the entire population of ovaries and can be characterized by the mean and standard deviation. The standard deviation expresses the dispersion of the distribution and can be seen as a measure of the biological variability. When determining the number of follicles in the ovary an accurate estimate of the mean and the standard deviation need to be derived. In this regard, appropriate sample sizes are vitally important for narrowing confidence intervals to acceptable levels [7].

Mean follicle numbers in mice are known to vary considerably between animals of the same strain [8], as well as between strains [9]. An example of this variability is reported in Myers *et al*., [8]; various research groups report divergent number of follicles within the same strain, showing evidence of biological variability, albeit using a variety of counting methods. The variability presented specifically in the C57Bl/6 mouse line in the same age groups is particularly remarkable. It is on the basis of this variability that Faddy and Gosden [7] emphasize the requirement for much larger datasets than those currently used, to assess properly the follicle numbers within ovaries of specific species, strain and age. Our work aims at assessing how the biological variability, i.e. dispersion of the distribution, affects counting experiments, and at assisting in choosing an adequate number of animals and sampling frequency; this will allow, on one hand, avoiding inaccurate follicle estimates and drawing ambiguous conclusions from underpowered studies, and on the other hand, reducing the unnecessary use of tissue and experimental workload.

We have developed computer algorithms to computationally generate mouse ovaries, based on spatial and morphological characteristics derived from measurements performed on actual ovarian sections of neonatal mice. Herein, we report how the deviation of the follicle-number estimates from their actual mean is affected by the number of ovaries and sampling frequency used.

## Methods and Algorithms

In this work we use the term ‘simulated’ ovary to indicate a computer model made of spheres in a 3-dimensional space, the size and the spatial distribution of which closely resemble those of follicles within a real ovary. More specifically, the sizes of these spherical structures are based on the average sizes of actual follicles measured on ovarian sections of C57Bl/6 mice, for different developmental stages [10 and 11], namely primordial, primary and secondary. Transitional follicles (the stage between primordial and primary) were not modelled in this work due to the fact that this stage is still equivocal among researchers, both from a morphological and a developmental point of view [10]. Furthermore, due to the fact that we are examining neonatal mouse ovaries, any mature follicles, i.e. from pre-antral stage onwards, were not modelled. The spherical structures in the simulated ovary contain co-centric spheres, corresponding to the oocyte, nucleus and nucleolus of the follicle. The sizes of the spheres are assigned based on experimental measurements which depend on the developmental stage of the follicles, the animal’s age as well as the species. It must be stressed that the simulated ovary is contingent on the experiment that needs to be simulated.

### Biological data

The raw data for the modelling were collected from published data of the neonatal mouse ovaries. Actual follicle number estimates were obtained from the data published by Kerr's group [12], presented in Table 1. Follicle sizes and spatial data were obtained by Da Silva-Buttkus *et al*., [11], for day 8 and day 12 ovarian sections of C57Bl/6 mice; follicle diameters and follicle distances from the ovarian epithelium wall are reported in Table 2.

**Table 1:** Average follicle numbers in whole neonatal C57Bl/6 mouse ovaries.

Age in days	Total primordial follicles (±*SEM*)	Total primary follicles (±*SEM*)	Total secondary follicles (±*SEM*)
7	1987 ± 203	569 ± 35	5 ± 4
12	2317 ± 289	362 ± 34	328 ± 34

Average follicle numbers in whole neonatal mouse ovaries (mean ± standard error of the mean): data reported by Kerr *et al*. [12]. 6 mice were used to estimate day 7 follicle numbers and 7 mice to estimate day 12 follicle numbers.

**Table 2:** Average follicle *diameter* and *distance* from ovarian epithelium wall.

Follicle Stage	Day 8		Day 12		
	Diameter (μm)±*SEM*	Distance (μm)±*SEM*	Diameter (μm) ± *SEM*		Distance (μm) ± *SEM*
Primordial	20 ± 5	30 ± 25	18 ± 5	24 ± 17	
Primary	48 ± 5	69 ± 29	51 ± 10	70 ± 29	
Secondary	–	–	79 ± 7	174 ± 77	

Average follicle *diameter* and *distance* from ovarian epithelium wall (mean ± standard error of the mean). Data from neonatal C57Bl/6 mouse ovarian section analysis [11].

We need to emphasise here two important issues. Firstly, we are using the follicle-number of day 7 mice (Table 1), although associating them to the spatial and size characteristics of day 8 mouse follicles (Table 2). Given the inherent variation in the time of birth and time of sacrifice for the neonatal mice, we assume that follicle distributions of day 8 mice are approximately similar to those of day 7 mice. Secondly, the follicle diameters, *D*_*f*_, were measured only on those follicles showing a clear sharp nucleus on the section, disregarding any follicles that had a fuzzy or imperceptible nuclear profile. The diameter is determined as the average between two perpendicular segments taken on the follicle profile (see methods in [11]). The diameters of the oocyte, *D*_*o*_, were additionally measured on ovarian sections from randomly selected follicles, which present an oocyte in their cross-sectional profile. The following diameter ratios were then calculated: *R*_*o-f*_ = *D*_*o*_/*D*_*f*_, where *D*_*o*_ and *D*_*f*_ are the average oocyte and follicle diameters, respectively; these ratios are developmental-stage dependent, as reported in Table 3, and were used to generate the simulated ovaries.

**Table 3:** Ratios between oocyte and the follicle diameter, *R*_o-f_.

Follicle Stage	*R*_*o-f*_ = *D*_*o*_ /*D*_*f*_
Primordial	0.78
Primary	0.64
Secondary	0.54

### Computer generation of simulated ovaries

The following subsections will illustrate how simulated ovaries are generated; the relative computer algorithms were implemented in Fortran 77, unless otherwise stated.

### Generating follicles numbers and diameters

In order to generate a simulated ovary of specific age we randomly select the number of follicles for each developmental stage. We assume that follicle numbers follow a Gaussian distribution, with mean and standard deviation reported in Table 1: a random number (*N*_*stage*_) of follicles is obtained for the given developmental stage. We then generate each follicle by randomly assigning to it a diameter. We assume that follicle diameters follow a Gaussian distribution, with mean and standard deviation reported in Table 2. The diameter of the oocyte for each follicle is assigned according to the ratio values reported in Table 3. This is repeated until *N*_*stage*_ follicles are generated.

### Inserting follicles into the simulated ovary

The process described in the previous subsection generates *N*_*tot_fol*_ = ∑ *N*_*stage*_ spheres of different sizes, which need to be inserted in a virtual spherical volume (the simulated ovary) without overlapping, and with a spatial arrangement typical of follicles in an actual ovary [11 and 13]. Firstly, the volume of the simulated ovary, *V*_*ovary*_, has to be selected. For this purpose, the total volume occupied by the follicles, *V*_*tot_fol*_, is calculated as the sum of the volumes of each follicle, $V_{fol}^{i}$:

$$V_{tot\_fol}=\sum_{i=1}^{N_{tot\_fol}}V_{fol}^{i}$$ (1)

For simplicity, the simulated ovary is assumed to be spherical, with a volume *V*_*ovary*_. The value to be assigned to *V*_*ovary*_ is calculated as in Eq. 2:

$$V_{\mathrm{ovary}}=\;{aV}_{\mathrm{tot\_fol}}\;\left(a\;>\;1\right)$$ (2)

where *a* is an arbitrary parameter (*a*>1), which is adjusted in order to accommodate all the follicles and to ensure that the simulated is realistic, as it is explained below.

In order to insert a follicle into the simulated ovary volume, a radial-direction of the ovary is randomly chosen (see page 111 of [14]). The follicle is placed along the chosen direction, at a distance from the ovarian wall randomly picked from a Gaussian distribution, with mean and standard deviation as reported in Table 2.

**Fig 1:**
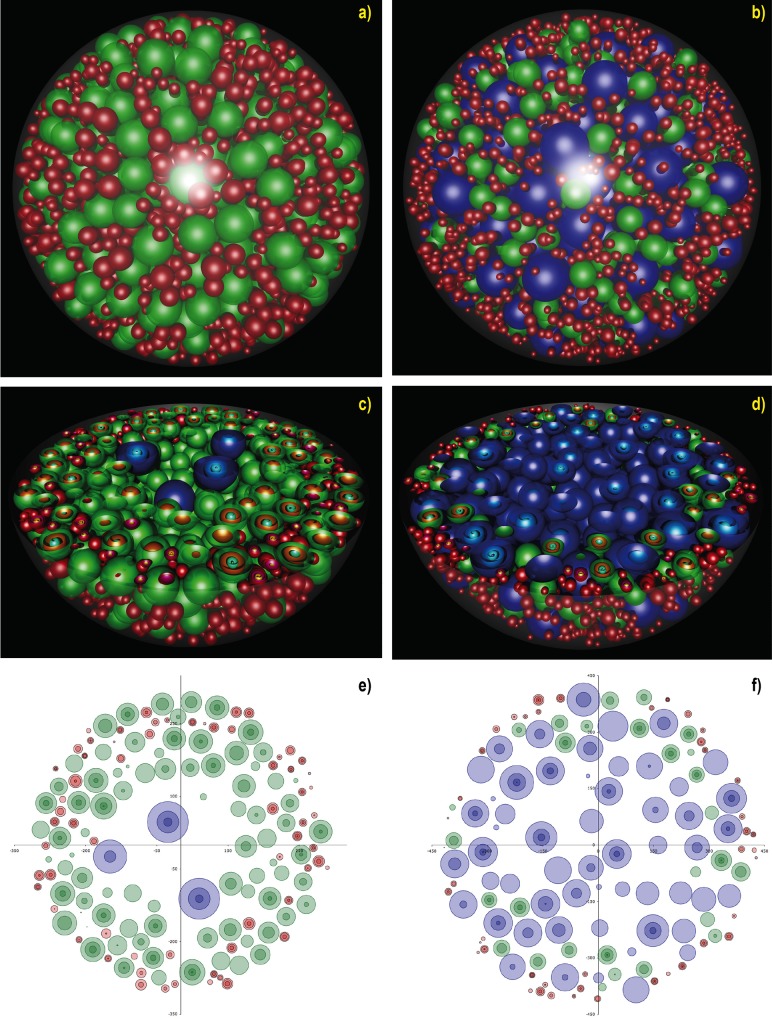
Local follicle distribution in the 3-dimensional simulated ovarian volume. 3-D renderings of day 8 and day 12 simulated ovaries, created using the Perspective of Vision Ray tracer (www.povray.org). Red spheres are primordial follicles, green are primary and blue are secondary follicles. Each follicle has a unique diameter, randomly selected from a specific distribution as explained in methods. Images a) and b) show the whole ovary (day 8 and day 12, respectively); the distribution of follicles inside the 3D volume of these ovaries is shown in the images c) (day 8) and d) (day 12). The resulting two-dimensional equatorial cross-sections are shown the images e) (day 8) and f) (day 12), drawn in MS Excel. Follicle numbers for Day 8 simulated ovary: 2058 Primordial, 768 Primary, 5 Secondary. Follicle numbers for Day 12 simulated ovary: 2245 Primordial, 428 Primary, 389 Secondary.

Once all the *N*_*tot_fol*_ follicles are inserted in the virtual spherical volume, the follicle profile density, ρ_*profile*_, is calculated on the equatorial section of the simulated ovary:

$$\rho_{\;\mathrm{profile}}=\sum_{i=1}^{N_{p}}A_{\mathrm{profile}}^{i}/A_{\mathrm{section}}$$ (3)

where *A*_*section*_ is the area of the equatorial ovarian section, *A* ^*i*^_*profile*_ is the area occupied by follicle-profile *i*, and *N*_*p*_ is the number of follicle profiles on an equatorial section (see also Fig. 1 below). The simulated ovary is accepted if the profile density is within 10% of the one measured from real sections (see Table 4 and Supporting Information in [11]); otherwise a new ovary is generated with a different choice of the parameter *a* (Eq. 2). Out of all the simulated ovaries we have generated in this work, the value of *a* was found to range between 2.3–2.6, in order to get a density within the specified 10% requirement. The overall procedure ensures that each simulated ovary is made of a number of follicles and spatial properties similar to those found in an actual ovarian section.

**Table 4:** Profile density, ρ_*profile*_, calculated from actual ovarian sections [11].

Ovary age	Actual ρ_*profile*_ (Eq. 3)
Day 8	0.47
Day 12	0.64

350 simulated ovaries of day 8 and 12 were generated to perform the analyses herein; this number of ovaries allows sampling adequately all the relevant Gaussian distributions mentioned above, and ensures realistic follicle numbers, diameters and spatial arrangement.

### Assessing errors on follicle-number estimates

The primary objective of this work is to assess the error when estimating follicle numbers in neonatal C57Bl/6 mouse ovaries. For this purpose, the simulated ovaries are computationally analysed in order to reproduce follicle counting experiments. The ovaries are virtually sectioned and their follicle-number is estimated by applying the unbiased stereological technique, more specifically the disector and the fractionator ([14, 15 and 16], see also S1 Supporting Information). The *in-silico* stereological technique produces estimates of follicle numbers, primordial or primary, for different sample sizes, *N*, and sampling frequencies, *f*. We use *f* = 1/5 (count 1 section out of 5), *f* = 1/20 and *f* = 1/50.

The unique advantage of the simulated ovaries is that the total number of follicles is *a priori* known; therefore, it can be used for a direct comparison with its estimates. We recall that we generated simulated ovaries with follicle numbers following a distribution of known mean, μ, and standard deviation σ. In this work we want to provide an *estimate* of the standard error of the mean, *SEM* ^*N*,*f*^ for the number of follicles, when using a sample of *N* ovaries and a sampling frequency *f*. This *SEM* ^*N*,*f*^ is effectively an estimate of the error of follicle numbers when performing a real counting experiment with *N* ovaries and sampling frequency *f*.

In order to estimate the *SEM* ^*N*,*f*^ with sufficient statistical power, we generated *L* samples, of *N* ovaries each. Each sample, *i*, provides a different sample mean, $m_{i}^{N,f}$ (the arithmetic average of the *N* estimates of the follicle-number). The $m_{i}^{N,f}$ themselves are values of a statistical variable following its own distribution with standard deviation $\sigma_{m}^{N,f}$. This $\sigma_{m}^{N,f}$ is exactly what we want to estimate. Note that it is impossible to calculate exactly $\sigma_{m}^{N,f}$, as this would require the entire population in the distribution to be taken into account; therefore we can only provide an estimate using the following formula:

$$\sigma_{m}^{N,f}∼s^{N,f}=\sqrt{\sum_{i=1}^{L}\frac{{\left(m_{i}^{N,f}-\mu \right)}^{2}}{L}}$$ (4)

In Eq. 4, μ is the mean originally used to generate Gaussian distribution of the follicle numbers in the simulated ovaries, which can be found in Table 1. We stress here that, since the true mean of the distribution is known, *L* is reported in the denominator of Eq. 4, rather than *L-1*, which is used commonly when analysing experimental data, where the true population mean is not known. Furthermore, if we were to use a low value of *L*, the *SEM* ^*N*,*f*^ would depend on the chosen set of simulated ovaries; to eliminate this dependence, we use *L* = 10,000 samples by applying the bootstrapping approach ([17], see also S2 Supporting Information). Samples of *N* ovaries (*N* = 1, 2, 3, 4 …, 20) are generated by picking, with replacement, from the pool of 350 ovaries. Each sample yields an estimate of the follicle number mean, $m_{i}^{N,f}$. Eq. 4 is applied to obtain an estimate of the standard error of the mean: *SEM* ^*N*,*f*^ ~ *s* ^*N*,*f*^. This is repeated for each of the 20 groups made of 10,000 samples of *N* ovaries. The bootstrapping procedure is recommended when the theoretical distribution of a statistical variable of interest is complicated [18], as in the case of the simulated ovary. Furthermore, if we were to generate simulated ovaries *on-the-fly*, we would need to generate 10,000×(1+2+3+…+20) = 2,100,000 ovaries for each age, which is impractical even for a computational procedure.

## Results

### Structure of the simulated ovary

**Fig 2:**
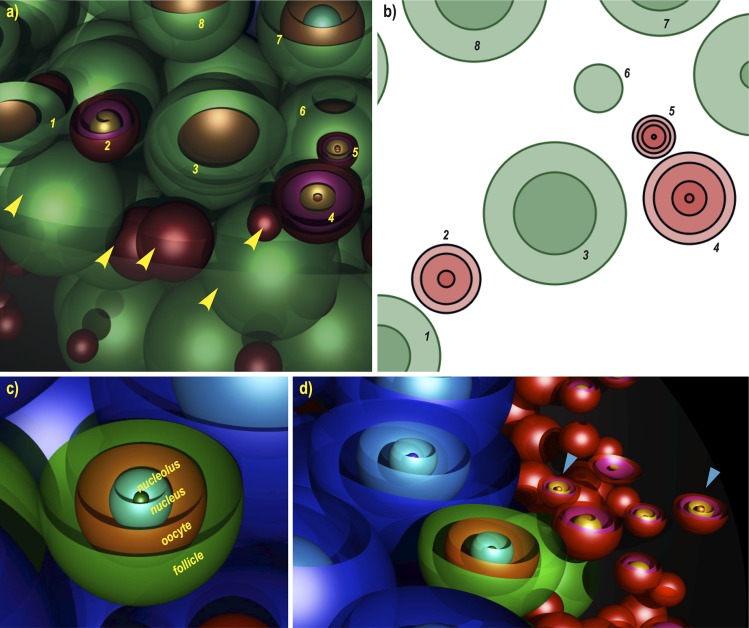
Details of 3D illustration of a simulated ovary. Part of a day 8 simulated ovary, a) and its corresponding cross section, b). Primordial (red) and primary (green) follicles numbered respectively in both a) and b) are virtually sectioned. Yellow arrows point at follicles in the immediate vicinity of the numbered ones, albeit not appearing in the 2D section. Oocyte, nucleus and nucleolus have been added to the model for illustrative purposes, c). These structures can also be simulated based on real measurements, in order to be used in the stereological counting. d) shows an example of a secondary, a primary and two primordials (blue arrows) with all internal profiles visible (day 12 ovary).

Fig. 1 illustrates two simulated ovaries produced using the algorithm described in the Methods and Algorithms section and visualised in 3D (using the Persistence of Vision Ray tracer; www.povray.org). In these illustrations the oocyte, nucleus and nucleolus of the follicles have been added, in order to show the internal appearance of the follicle profiles upon sectioning (Figs. 1 and 2). In Fig. 1C and 1D, the hemispheres (virtual half sectioning) show the internal distribution of the follicles in three dimensions; the corresponding two dimensional cross-sections produced from the same location of the simulated ovaries are shown in Fig. 1E and 1F. Fig. 2A and 2B present a ‘cortical’ area close to the edge of a day 8 ovary, where the follicle density is high. Fig. 2C shows internal structures, i.e. oocyte nucleus and nucleolus, for illustrative purposes. Fig. 2D shows a secondary, a primary and two primordial follicles with all internal structures visible (day 12 ovary).

### Estimating the Standard Error of the Mean (*SEM*)

The *in-silico* unbiased stereological technique for estimating numbers of follicles is applied to the simulated ovaries. *L* = 10,000 samples, each containing *N* ovaries, are chosen and for each sample a follicle-number mean, $m_{i}^{N,f}$, is estimated using a specific sampling frequency, *f*. The standard error of the mean, *SEM* ^*N*,*f*^, is estimated according to Eq. 4, for different sample sizes (namely *N* = 1, 2, 3, 4 …, 20 ovaries), for different counting frequencies (*f* = 1/5, *f* = 1/20 and *f* = 1/50), different ages and follicle stages.

**Fig 3:**
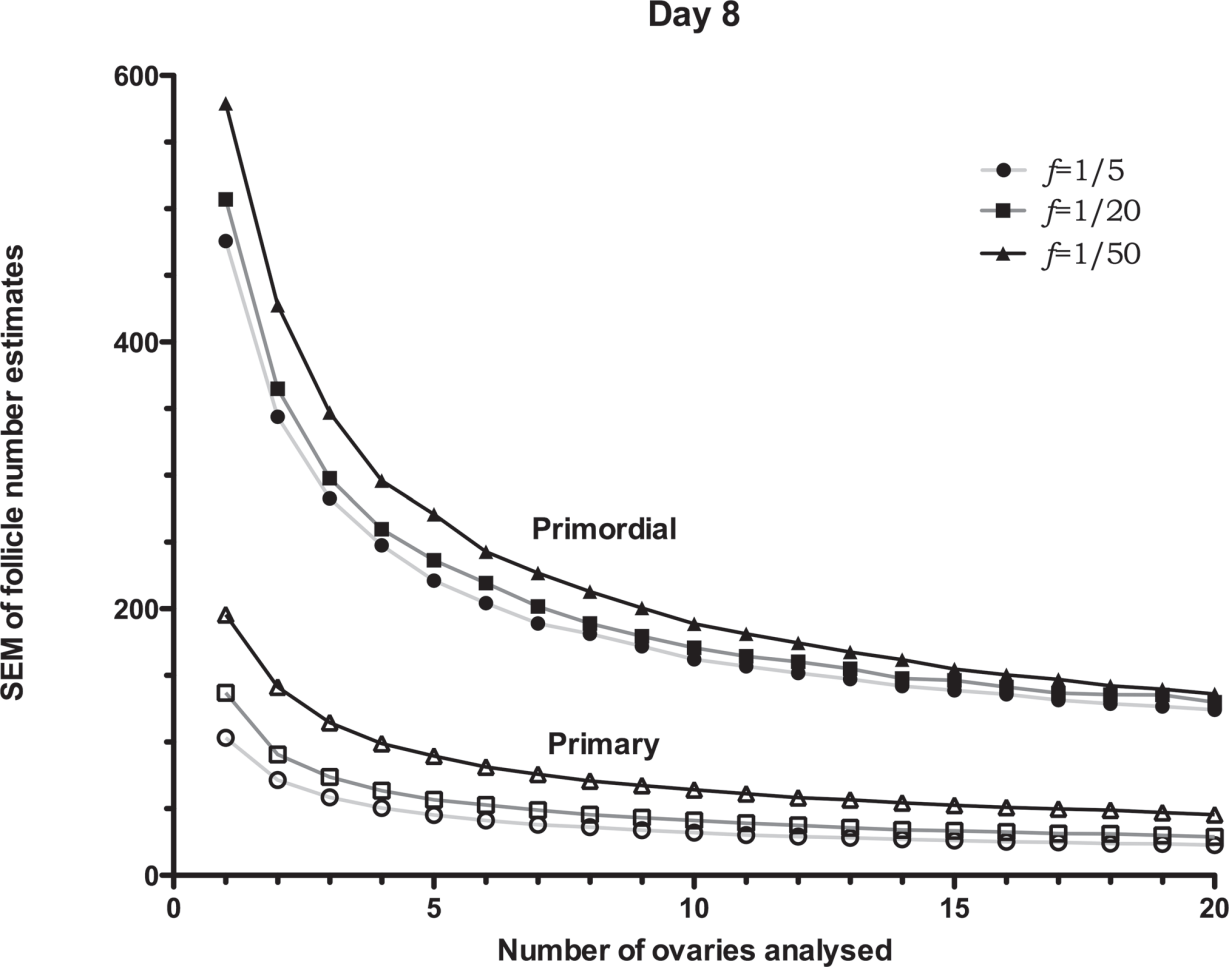
Day 8 *SEM*s of follicle-number estimates. *SEM*s of follicle-number estimates for day 8 simulated ovaries. The *SEM*s, calculated using 10,000 samples, are reported for different number of ovaries (*N* = 1, 2 … and 20 ovaries), and sampling frequencies, *f*. *SEM*s are reported for primordials (filled symbols) and primaries (non-filled symbols). Distribution properties (μ±*SEM*): Primordial: 2000±203, Primary: 570±35 (see Table 1).

Fig. 3 reports the *SEM*s of day 8 mouse ovaries for primordial and primary follicles; as expected, Fig. 3 shows a decreasing trend in the *SEM* as the number of ovaries used increases. For instance, the graph shows that in the case in which two ovaries are used and *f* = 1/5, the *SEM* for the primordial follicle number is equal to 344 follicles, which corresponds to a relative deviation (*SEM*/mean) of 17% (given that the mean number of primordial follicles is 2000), whereas, for 10 ovaries, the relative deviation (*SEM*/mean) drops to around 8%. It is, also, interesting to notice how the sampling frequency affects the *SEM*s. In the case in which two ovaries are used, the *SEM* for the primordial follicle-number is equal to 364 follicles for *f* = 1/20 (*SEM*/mean = 18.2%), while for *f* = 1/50, *SEM* = 428 (*SEM*/mean = 21%), which corresponds to an extra error of 64 follicles.

As expected, a similar trend is obtained when counting primary follicles in day 8 ovaries, (Fig. 3); in fact, the *SEM* decreases as the sample size increases. Indicatively, *SEM*/mean = 13% when using two ovaries and a sampling frequency *f* = 1/5, which drops to 8% when using 10 ovaries and *f* = 1/50. A further drop to 6% occurs when the frequency is increased to *f* = 1/5, which is negligible considering the significant increase in lab work to achieve this.

**Fig 4:**
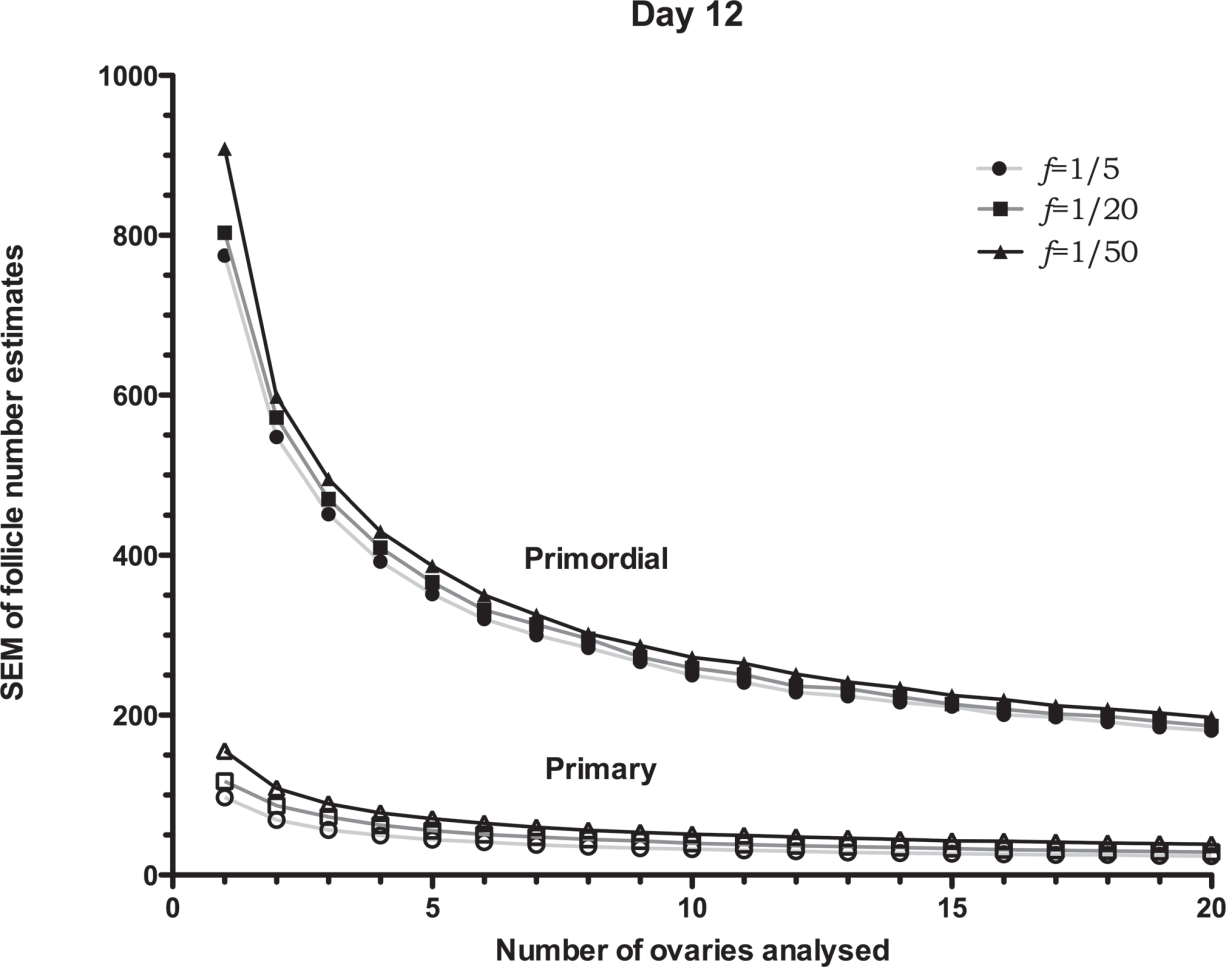
Day 8 *SEM*s of follicle-number estimates. *SEM*s of follicle-number estimates for day 12 simulated ovaries. The *SEM*s, calculated using 10,000 samples, are reported for different number of ovaries (*N* = 1, 2 … and 20 ovaries) and sampling frequencies, *f*. *SEM*s are reported for primordials (filled symbols) and primaries (non-filled symbols). Distribution properties (μ±*SEM*). Primordials: 2300±289, primaries: 360±34 (see Table 1).

The *SEM*s for day 12 ovaries are reported in Fig. 4, in agreement with those of day 8; as the sample size increases, the error on the estimates decreases. Due to the slightly increased standard deviation in the average number of follicles in the day 12 ovary compared to the day 8 (Table 1), the *SEM*s reported are greater, especially for smaller sample sizes. Interestingly, Fig. 4 shows that if four or less ovaries are used to estimate the number of primordial follicles in a day 12 mouse, regardless of the sampling frequency, there would be an error of around 400 follicles, a finding which is quite significant when compared with the average follicle number, 2300. Errors of this magnitude may be crucial when performing experiments to distinguish two follicle-number distributions, when the difference may be due to developmental age, effects from drugs or radiation, or the effect of a mutation. A potential application of the simulated ovary would be to investigate the accuracy of the follicle number estimates in a mouse ovary where the follicle number is significantly different from a control.

## Discussion and Conclusions

The accurate estimation of follicle numbers in mammalian ovaries is a crucial and still challenging task in the field of reproductive biology [19]. The accuracy of these estimates is affected by two factors: the biological variability within a given mouse line and the frequency used to sample the ovary. A quantitative approach has been presented in this work, which simulates mouse ovaries of a specific age and strain and reproduces counting experiments *in-silico*. This approach can be applied for ovaries of any mammal, provided that minimal information about follicle-number distributions and spatial arrangement are available. The simulated ovary is a computational tool designated to assist the investigator in improving efficiency when performing counting experiments, both in terms of use of tissue and laboratory time.

We have presented quantitatively how the accuracy in estimating the mean follicle number is affected by varying the number of ovaries and choosing different counting frequency—Figs. 3 and 4. Those figures can be used for better experiment planning. For instance, while on one hand, a high number of ovaries may be required to reduce the error down to a given threshold, on the other hand, our work shows how to reduce the number of sections analysed, which may result in an overall less labour-intensive experiment. As an example, if an accuracy of 10% is required (error of around 200 follicles for a population with a mean of 2000), at least 6 ovaries must be used (Fig. 3), but the sampling frequency needs to be high, *f* = 1/5, i.e. count 1 section out of 5. Using *f* = 1/50, rather than *f* = 1/5 would require 9 ovaries (Fig. 3), but would significantly reduce the ovarian sections to count, and therefore lab time, as fewer sections in total would be required. In order to appreciate the reduction in labour time, let’s assume a set of neonatal mouse ovaries of 600μm diameter, containing 2000 follicles on average. Selecting a section thickness of 5μm, each ovary would produce approximately 120 serially-cut sections. Referring to Fig. 3, estimating the follicle number with an error of 200 follicles, one can either sample 10 ovaries counting one every 5 sections, or use 15 ovaries counting one every 50 sections. In the first case, 240 sections would be sampled, whereas in the latter, only 36 sections would be required to reach a similar accuracy.

It is interesting to compare the *SEM* results in Figs. 3 and 4 to the experimental results by Kerr *et al*. [12], also reported in Table 1. In Fig. 3 the *SEM* for primordial follicles is 204 (6 ovaries and *f* = 1/5), while the experimental *SEM* is 203; for primary follicles the *SEM* from Fig. 3 is 41, while the experimental *SEM* is 35. For day 12, Fig. 4, the *SEM* for primordial follicles is 300 (7 ovaries and *f* = 1/5), while the experimental *SEM* is 289; for primary follicles the SEM from Fig. 4 is 37, while the experimental *SEM* is 34. The close match between the calculated and experimental *SEM*s corroborates the validity of the simulated ovary.

Sometimes, a caveat for a sufficient number of animals is the challenge to harvest them; mice need to be bred, which can be expensive and time-consuming. Furthermore, if the study involves a mutation that affects the health of the mouse, as well as fertility, it may be difficult to collect a large number of ovaries. In those cases in which the number of ovaries cannot be chosen arbitrary, the simulated ovary can provide insight on how a higher a number of sections can improve the counting accuracy. This applies particularly to experiments performed using human ovaries, which are extremely rare and challenging to obtain (e.g. the data set in [20]). Therefore, careful experiment planning and best use of tissue is of strategic importance to ensure that follicle numbers are estimated with the required accuracy. Hence, the proposed approach can be applied in the planning phase, to estimate the requirements in terms of tissue and laboratory time; it may well be that the higher time demand in using more ovaries can be offset by lowering the sampling frequency.

Finally, the simulated ovary approach can be adapted for and applied to other organs characterised by a large number of individual functional units (e.g. neuronal and glial cells in the brain). In fact, the study of the development of these organs, or the effects of toxins, radiation exposure, environment or genes on their function, directly relates to the accurate counting of their functional units.

## Supporting Information

S1 Supporting Information Application of the unbiased stereological technique. (DOCX)

S2 Supporting Information Schematic of bootstrapping approach. Schematic representation of the bootstrapping approach for generating random samples of simulated ovaries.
(DOCX)
