# Supplementary material for: Computer-Generated Ovaries to Assist Follicle Counting Experiments
Source: PLoS One. 2015 Mar 26;10(3):e0120242. doi: 10.1371/journal.pone.0120242 (PMC4374836; doi:10.1371/journal.pone.0120242)
Supplement: S1 Supporting Information — (DOCX) [file pone.0120242.s001.docx]

# S1 Supporting Information: Application of the unbiased stereological technique

In this work we want to assess the accuracy of a simple unbiased stereological technique [15 and 16], which combines the fractionator scheme and disector method [14] in counting follicles within the mouse ovary. A simple implementation would involve serially sectioning the object (ovary). A sampling frequency, *f*, and a random starting point are chosen, which provide a sample of sections of the whole ovary. Every particle (follicle) in the sampled sections is counted (raw count), and then an unbiased estimate of the total number of the particles in the object is obtained by multiplying the raw count by the reciprocal of the frequency, 1/*f*. In order to properly count the particles within a sampled section (called the reference) the disector method needs to be applied. In this method the adjacent section (called look-up) to the reference one is considered. A particle is counted only if the particle’s profile (cross-section) is present only in the reference section and not in the look-up section. A simple example of the disector method is illustrated in figure A1. Further details and applications of the unbiased techniques can be found in [14].


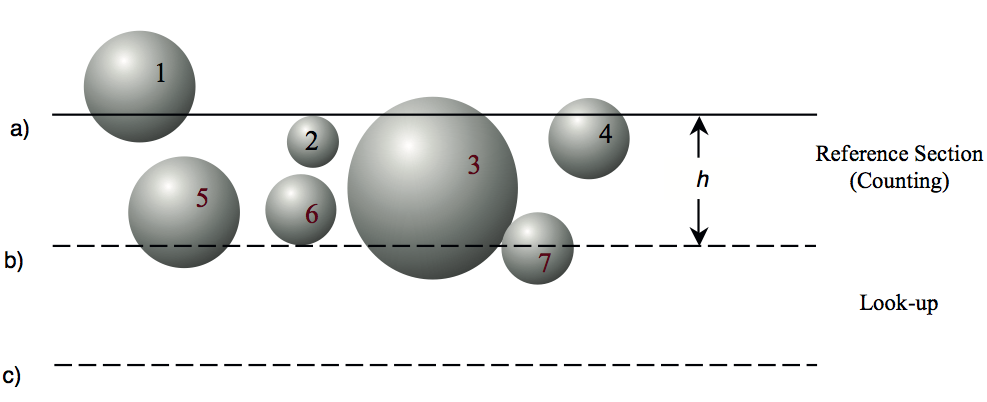


**Figure A.1**: **Application of the unbiased stereological technique.** Assume two sections of an object containing spherical particles. The sections are cut at specific thickness *h*. The particles that would be counted are those transected only by the reference section and not by the look-up section. In the present example, spheres 1, 2 and 4 would be counted, transected by plane a) and not by b) and c), i.e. appearing only in the reference section. Spheres 3, 5, 6 and 7 would not be counted, as they are either cut by plane b) or not cut at all (sphere 6).

A Fortran algorithm has been developed to apply this stereological technique to the simulated ovaries. The algorithm virtually cuts through the ovary, producing sections of given thickness. The ovarian section thickness considered for this work was 5µm since this value is routinely used in various labs. The frequency of the counting can also be chosen. The disector counting technique is then applied to the sampled sections, starting from a random section. For example, let’s assume that the user selects a thickness of 5µm and a counting frequency of every 10 sections, for a 500µm diameter ovary (100 sections in total). The algorithm will start from a random section between the 1st and 10th, e.g. the 7th. Then the sections considered will be the 7th, 17th, 27th ... and 97th. For each sampled section, the program will count the profiles of follicles (one can also use oocytes, nuclei or nucleoli, if measurements for modelling are available) appearing on top of the reference section but not appearing on the top of the adjacent section (look-up section). In this way, a raw count of the follicles (different for each developmental stage) is produced. To produce an estimate of the actual number of follicles in the whole ovary, the raw count is multiplied by the reciprocal of the sampling frequency. In the case of the example, the raw count is multiplied by 10. This method provides an unbiased estimate of the total number of follicles in the ovary.
