# Supplementary figures and images for: Computer-Generated Ovaries to Assist Follicle Counting Experiments
Source: PLoS One. 2015 Mar 26;10(3):e0120242. doi: 10.1371/journal.pone.0120242 (PMC4374836; doi:10.1371/journal.pone.0120242)

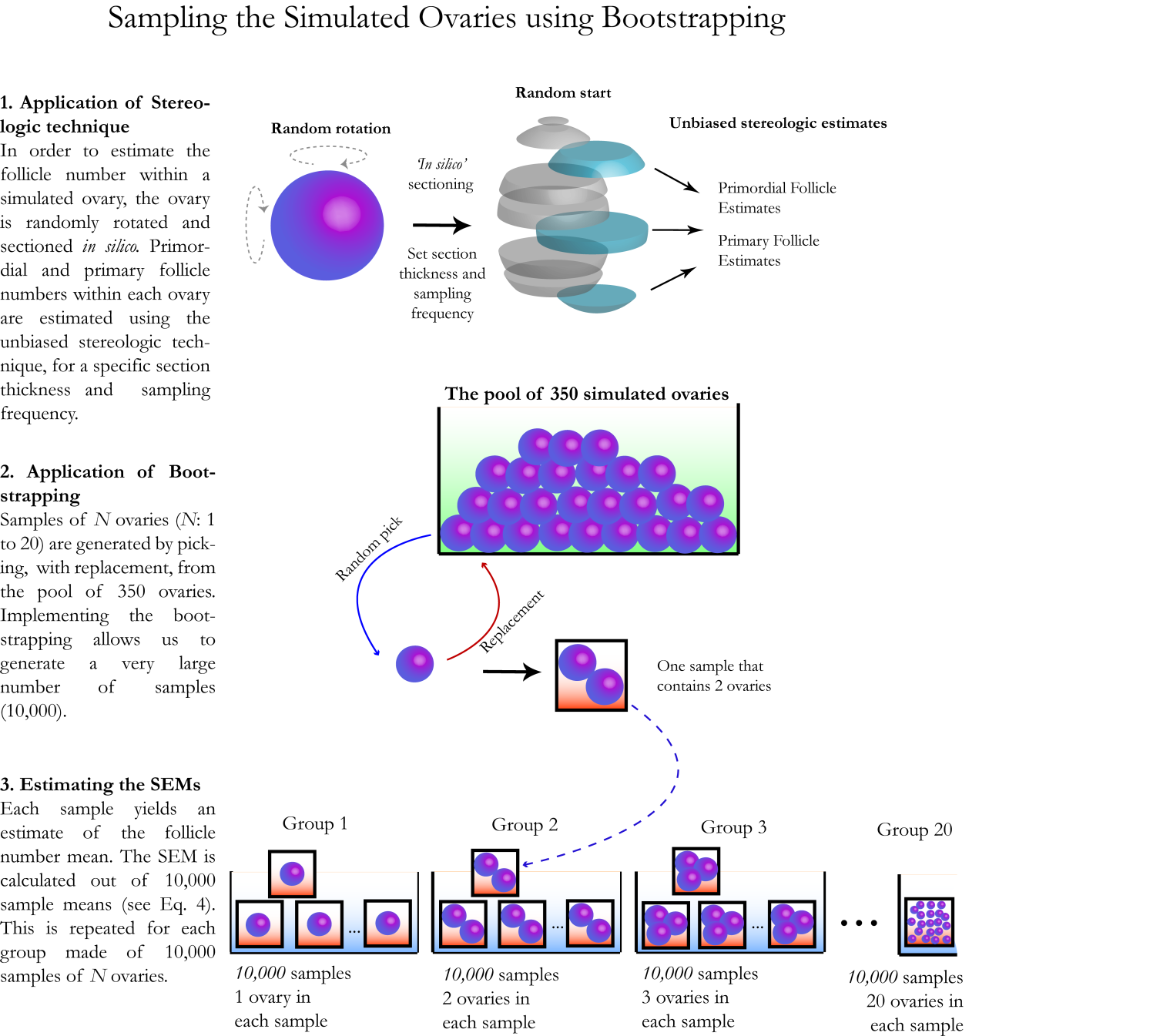

Supplement: S2 Supporting Information — Schematic representation of the bootstrapping approach for generating random samples of simulated ovaries. (DOCX) [file pone.0120242.s002.docx]
